# Welfare and Labour

**Published:** 1920-08-21

**Authors:** 


### Welfare and Labour.

The abolition of half-time labour for schoolboys whose education and general welfare have been sacrificed to industry is not only a step of educational advantage, but also of social and industrial improvement. The Factory Acts and other measures have ensured some sort of minimum of decency and safety, and the withdrawal of schoolboys is in reality part of the Welfare movement. In a recent paper, Miss Voysey (General Secretary of the Welfare Workers' Institute, London) dealt with this subject from the point of view of getting voluntarily a better and more human interest into the workshops, and what she said seemed to explain to some extent the suspicion with which many work

THE PUBLIC WELL-BEING—(*continued*).

men have viewed welfare work. The outbreak of war interrupted the normal course of development of this movement, and for a time diverted it from its proper channels. The need for immediate results and for greatly increased production over-emphasised its material side, and there was some confusion of ideas. Workers were inclined to look upon it as a device to get more work out of them, and there has been difficulty in breaking down that suspicion, especially as there are not lacking many who do their best to prevent any development of better mutual understanding between employers and employed. We are convinced that the suspicion is not well-founded, and that welfare work started in the many great firms is disinterested. It is a sign that modern industry no longer imagines that men are mere machines of production, but realises that they are human beings whose working conditions should be put upon the best possible basis.

It is a fundamental error that regards work as a disagreeable necessity, and considers that the less there is of it for the individual the better it is for him. It is a far greater mistake to ignore every chance of improving its physical conditions, and labour might have done essentially better for itself if upon that it had concentrated some of the energy devoted to wages and hours. Employers have done a great deal in the way of recreation grounds, sports clubs, libraries, etc., and have found cordial co-operation. Some great undertakings have created model communities for their workers, and a great many firms have welfare supervisors who can, if they are so allowed, exercise a thoroughly beneficial influence all round. Why does not labour use these opportunities whole-heartedly, and get a little joy into work, and some return to pride in labour? To view labour as economics served neat is a fault that employers made in the past, and organised workers are in danger of repeating it now. Labour should give even the devil his due, and make the best of him. Those who refuse because they dislike the present system of industry are cutting off their noses to spite their faces. Labour has a duty to itself which it is far from performing yet, and it has given little or no attention to the general question of national health. It should be its close concern, and if it would spare time for constructive propaganda in that direction it would be doing a national service in addition to serving its own ends. It must co-operate as well as criticise, and it can probably do more than any other section of the community in this individual matter of public health. It also stands to gain more from it than any other section. It would be a good beginning to help in making the welfare movement what it is intended to be.

